# Entropy of the Quantum–Classical Interface: A Potential Metric for Security

**DOI:** 10.3390/e27050517

**Published:** 2025-05-12

**Authors:** Sarah Chehade, Joel A. Dawson, Stacy Prowell, Ali Passian

**Affiliations:** 1 Quantum Information Science Section, Oak Ridge National Laboratory, Oak Ridge, TN 37830, USA; passianan@ornl.gov; 2 Computational Sciences and Engineering Division, Oak Ridge National Laboratory, Oak Ridge, TN 37830, USA; 3 Resilient Cyber-Physical Systems Section, Oak Ridge National Laboratory, Oak Ridge, TN 37830, USA; dawsonja@ornl.gov (J.A.D.); prowellsj@ornl.gov (S.P.); 4 Cyber Resilience and Intelligence Division, Oak Ridge National Laboratory, Oak Ridge, TN 37830, USA

**Keywords:** entropy, quantum security, quantum–classical interface, risk assessment, entropic measures, quantum information theory, classical information theory

## Abstract

Hybrid quantum–classical systems are emerging as key platforms in quantum computing, sensing, and communication technologies, but the quantum–classical interface (QCI)—the boundary enabling these systems—introduces unique and largely unexplored security vulnerabilities. This position paper proposes using entropy-based metrics to monitor and enhance security, specifically at the QCI. We present a theoretical security outline that leverages well-established information-theoretic entropy measures, such as Shannon entropy, von Neumann entropy, and quantum relative entropy, to detect anomalous behaviors and potential breaches at the QCI. By linking entropy fluctuations to scenarios of practical relevance—including quantum key distribution, quantum sensing, and hybrid control systems—we promote the potential value and applicability of entropy-based security monitoring. While explicitly acknowledging practical limitations and theoretical assumptions, we argue that entropy-based metrics provide a complementary approach to existing security methods, inviting further empirical studies and theoretical refinements that can strengthen future quantum technologies.

## 1. Introduction

The rapid advancement of quantum technologies has highlighted the significance of the quantum–classical interface (QCI), a critical “node” where quantum and classical systems interact and communicate. Although the QCI is vital for leveraging quantum technologies, it also introduces unique security vulnerabilities. Historically, the boundary between quantum and classical realms has remained distinct [1] and largely unexplored. An example of how classical signals influence quantum-state coherence at QCIs was recently reported based on a Lindblad master equation approach [2]. As hybrid quantum–classical systems become more prevalent, the traditional notions of information and security warrant closer examination given that quantum information fundamentally differs from its classical counterpart, a disparity that introduces complex challenges in securing the information flux across the QCI. In particular, entropy quantifies uncertainty, which is crucial in security applications. Different entropy measures ensure various security aspects such as average uncertainty, conditional uncertainty, and distinguishability.

Central to our position paper is the introduction of entropy as a metric for QCI security. We aim to explore how entropy, as a measure of uncertainty, can help quantify information exchange between quantum and classical domains, revealing potential vulnerabilities and guiding protective strategies. To fully realize this approach, it is of key importance to map the intricacies of quantum systems, their interaction with classical control mechanisms, and the impact of quantum measurements [3] and external perturbations [4]. Our aim is to stimulate methods that quantify the information interchange between the quantum and classical spheres, offering information on possible vulnerabilities and charting pathways for protection of quantum devices.

The study of the quantum–classical interface (QCI) and hybrid quantum–classical systems remains open, lacking a universally accepted framework. This manuscript does not claim to provide a definitive formulation but rather presents a structured perspective to inspire further exploration. While many studies focus on the structural and dynamical consistency of hybrid systems [5,6,7], our motivation stems from security concerns—specifically, whether the QCI introduces vulnerabilities that can be quantified using entropy-based methods. Given the challenges of hybrid dynamics, alternative formulations may be more suitable in different contexts [5,7]. We argue that alongside fundamental studies, security implications must be considered, especially in applications involving quantum sensors, cryptography, or hybrid control. By refining interface conditions and leveraging entropy, we offer a perspective that may guide both theoretical and experimental investigations into security risks in hybrid systems. Ultimately, this work aims to stimulate further discussion and refinement rather than propose a final or exhaustive framework.

Our presentation is organized as follows: In Section 4, we discuss classical, quantum, and quantum–classical interfaces and present a formal definition of the QCI. Section 5 highlights all the proposed entropic functions that evaluate QCI, while Section 6 proposes the use of complementary metrics that are common anomaly detectors. Section 7 outlines reasonable criteria for QCI security and highlights known classical counterparts. We end this position paper with a discussion in Section 8 and concluding remarks in Section 9.

## 2. Hybrid Quantum–Classical Dynamics

Modeling hybrid systems involving quantum and classical degrees of freedom is central to QCI security. Several important methodologies have emerged to address these hybrid systems in practice. Among the most prominent approaches are path integral methods, surface-hopping techniques, quantum–classical bracket formulations, and statistical moment-based methods, which we describe briefly.

Path integral methods incorporate Feynman’s formalism to address quantum coherence and decoherence effects. A key example is the quantum–classical path integral (QCPI) method, which treats quantum subsystem dynamics interacting with classical environments via influence functionals and coupled quantum–classical paths. This allows for the detailed modeling of quantum nonlocality and memory effects [8,9].

Surface-hopping methods, typified by Tully’s fewest-switches surface-hopping (FSSH) algorithm, offer intuitive and practical tools for simulating nonadiabatic transitions between quantum states. These methods propagate classical trajectories on electronic potential energy surfaces and allow probabilistic transitions (hops) between surfaces to approximate quantum-state dynamics. They have been proven to be particularly effective in photochemistry and electron transfer [10,11].

Quantum–classical bracket approaches employ hybrid brackets combining quantum commutators and classical Poisson brackets, exemplified by the quantum–classical Liouville equation (QCLE) developed by Kapral and Ciccotti. QCLE provides a formal and consistent basis for mixed quantum–classical propagation, ensuring the proper conservation of energy and accurate limiting behaviors for purely quantum or classical dynamics [5,12,13,14].

Moment-based hybrid dynamics methods represent an innovative approach, describing the quantum–classical system using statistical moments (e.g., expectation values, variances). The hybrid moment formalism systematically captures quantum back-reactions on classical variables through hierarchical moment expansions and provides a consistent analysis of uncertainty propagation and quantum–classical interactions [1].

Each of these methods addresses quantum–classical interactions from distinct perspectives and therefore offer complementary strengths. The entropy-based approach introduced in this position paper could leverage these established methodologies. Conversely, QCI security considerations may motivate the extension of existing methods and enrich hybrid quantum–classical modeling.

## 3. Hybrid Equations of Motion

Deriving *hybrid equations of motion* is needed to describe the dynamical laws governing systems composed of interacting classical and quantum degrees of freedom, which possess a single or multiple QCIs. Formulating such hybrid dynamics consistently is however nontrivial because quantum and classical variables obey fundamentally different algebraic structures (commutators versus Poisson brackets) [5,15]. Various approaches introduce a *hybrid Hamiltonian*, combining classical and quantum parts, and a generalized equation of motion reducing correctly to classical or quantum limits in the appropriate scenarios [5,13].

Classical variables (q,p) evolve under Hamilton’s equations derived from the total Hamiltonian $H_{\mathrm{tot}}$, including the following interaction terms:

(1) $${\dot{q}}_{i}=\frac{\partial H_{\mathrm{tot}}}{\partial p_{i}},\;{\dot{p}}_{i}=-\frac{\partial H_{\mathrm{tot}}}{\partial q_{i}},$$

where quantum back-reactions through the expectation values of quantum operators are included, which effectively generalize Ehrenfest’s theorem to hybrid systems [16,17]. The quantum subsystem thus influences the classical subsystem via expectation-value forces, ensuring that classical trajectories are shaped by quantum averages rather than direct quantum uncertainties.

Quantum operators evolve according to the Heisenberg picture, with equations of motion governed by the total Hamiltonian:

(2) $$\frac{d\hat{O}}{dt}=\frac{1}{i\hbar }\left[\hat{O},H_{\mathrm{tot}}\left(q\left(t\right),p\left(t\right),\hat{O}\right)\right],$$

where classical variables (q,p) serve as time-dependent external parameters that influence quantum dynamics parametrically [1,13]. This parametric classical-to-quantum coupling is consistent with standard practices in quantum control and hybrid dynamics.

The hybrid equations presented in our work will not be derived from mean-field approximations nor from infinite-time decoherence limits. Instead, our formulation assumes a pragmatic, expectation-value coupling scheme as its principle, aligning with practical hybrid dynamics [5,16,17]. Moreover, our approach does not assume adiabatic evolution, thus permitting applicability to scenarios involving rapid or dynamically evolving quantum–classical interactions. Consequently, while our method relies on averaged quantum influences (thus inherently approximating quantum coherence effects), it remains broadly valid within controlled approximation regimes commonly used in hybrid quantum–classical treatments [1,13,17] and adequately serves the positional nature of our discussions. The presented arguments therefore provide context and justification for our chosen hybrid dynamics approach, which is within established practices in quantum–classical dynamics [1,5,15,16,17].

## 4. Preliminaries

### 4.1. Formalization of an Interface

In the QCI context, we define an *interface* as a condition under which the states of the two systems are coupled or connected to various degrees of strength. Let $u_{1}\left(p_{1}\right)$ and $u_{2}\left(p_{2}\right)$ represent the states of System 1 and System 2, respectively. Here, $u_{1}$ lies in a classical phase space if System 1 is classical, and $u_{2}$ lies in a quantum Hilbert space if System 2 is quantum. Thus, the state $u_{i}$ can be functions, vectors, or operators, depending on the nature of the system. The variables $p_{1}$ and $p_{2}$ represent the parameters specific to each system. For example, they could parameterize the state but do not define their evolution. To characterize the interaction between $u_{1}$ and $u_{2}$ over the parameters $p_{1}$ and $p_{2}$, we define a coupling function $K(u_{1},u_{2};p_{1},p_{2})$. Namely, the *interface condition* can then be expressed as an equation or constraint that satisfies $K(u_{1},u_{2};p_{1},p_{2})=0$ (or within a bounded tolerance). We then define a general interface equation:

(3) $$K\left(u_{1}\left(p_{1}\right),u_{2}\left(p_{2}\right)\right)=f\left(u_{1}\left(p_{1}\right)\right)-g\left(u_{2}\left(p_{2}\right)\right)=0,$$

where *f* and *g* are transformations or mappings that bring the states $u_{1}$ and $u_{2}$ into a common interaction space (e.g., converting quantum states to classical observables or vice versa).

In a control interface, the classical system often acts as an external controller, which modifies the quantum observable. To introduce a functional dependence in the transformation maps, such that the quantum state transformation depends on the classical states, we require

(4) $$K\left(u_{1}\left(p_{1}\right),u_{2}\left(p_{2}\right)\right)=f\left(u_{1}\left(p_{1}\right),u_{2}\left(p_{2}\right)\right)-g\left(u_{2}\left(p_{2}\right)\right)=0.$$

This modification allows a classical state $u_{1}\left(p_{1}\right)$ to influence the transformation function *f*, rather than assuming a fixed change.

If the systems evolve over time, the coupling depends on time *t*:

(5) $$K(u_{1}\left(p_{1}\right),u_{2}\left(p_{2}\right),t)=0,$$

which represents dynamic coupling, such as energy exchange or information transfer, where K changes over time based on the state evolution of each system.

**Example 1.**  *If $u_{1}$ is a quantum state |ψ〉 and $u_{2}$ is a classical observable x, then we define an interface by the expectation value:*

(6) $$K\left(u_{1},u_{2};p_{1},p_{2}\right)=\langle \psi |\hat{O}|\psi \rangle -x\left(p_{2}\right)=0,$$

*where $\hat{O}$ is a quantum operator for which its expectation value matches the value of the classical observable x after the measurement (x acting on the phase space). This coupling creates a link between the quantum and classical systems. In this example, x is the classical state variable representing an observed quantity in the system.*

**Figure 1:**
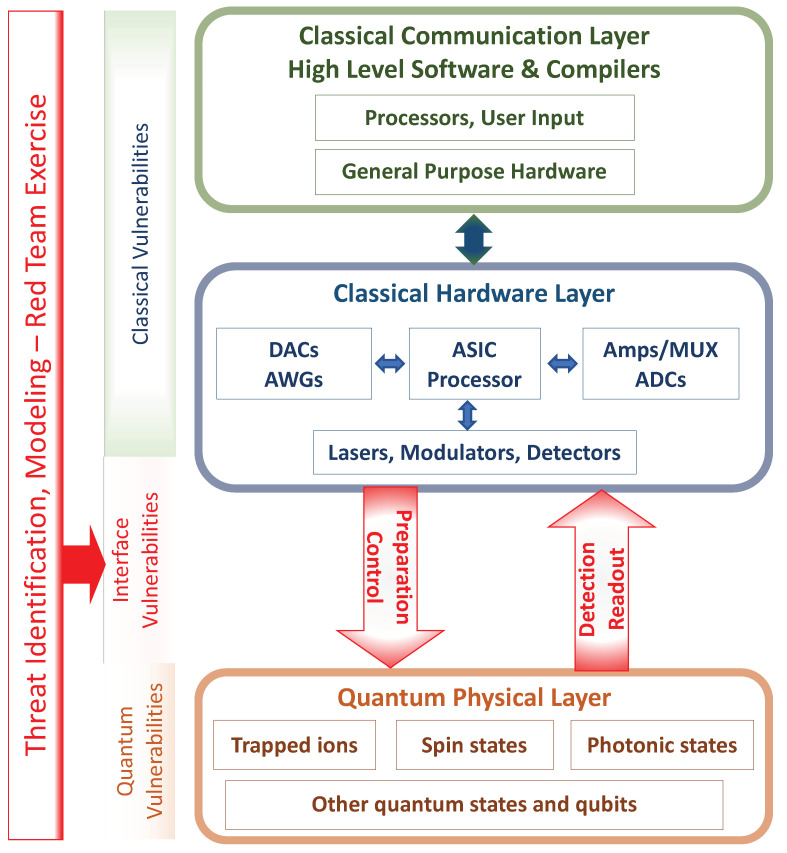
High-level depiction of a physical QCI in the context of a larger quantum information hardware stack. The conversion of classical physical states into discrete physical qubits—and vice versa—is enabled by hardware that, in effect, responds to control inputs encoded in logical bits or qubits.

This level of formal representation suffices for our objectives in the rest of this manuscript, and Figure 1 depicts the workflow of our QCI representation.

### 4.2. Interface from a Hamiltonian

Writing the interface equation in terms of Hamiltonians ensures that the evolution respects the underlying symmetries and conservation laws, and many hybrid quantum–classical models naturally arise from a Hamiltonian framework. To carry this out, we introduce the quantum Hamiltonian $H_{q}$, governing the quantum subsystem and classical Hamiltonian $H_{c}$, which governs the classical subsystem. The coupling term between the quantum and classical components can be incorporated into a total Hamiltonian $H_{\mathrm{tot}}$ defined as the total Hamiltonian of a generic QCI:

(7) $$H_{\mathrm{tot}}\left(q,p,\hat{O}\right)=H_{c}\left(q,p\right)+H_{q}\left(\hat{O}\right)+H_{\mathrm{int}}\left(q,p,\hat{O}\right),$$

where $H_{\mathrm{int}}\left(q,p,\hat{O}\right)$ mediates the interaction between the two subsystems. By considering the Hamiltonian equations of motion, we can develop an interface condition:

(8) $$C\left(q,p,\hat{O}\right)=-\frac{\partial H_{\mathrm{int}}}{\partial q}-\langle \frac{\delta H_{\mathrm{int}}}{\delta \hat{O}}\rangle =0.$$

That is, the classical subsystem is influenced by the quantum expectation values while maintaining consistency with the quantum commutator evolution. The first term $-\frac{\partial H_{\mathrm{int}}}{\partial q}$ represents the force exerted by the interaction Hamiltonian on the classical system. The second term $\langle \frac{\delta H_{\mathrm{int}}}{\delta \hat{O}}\rangle$ captures the quantum back-action on the classical system, where the variation with respect to $\hat{O}$ determines how the quantum state influences the evolution of the classical degrees of freedom. The functional derivative appears because $H_{\mathrm{int}}$ generally depends on quantum observables, which are operator-valued quantities, requiring a variational treatment to correctly describe their effect on the classical equations of motion. This term ensures that the expectation value of quantum fluctuations modifies the classical dynamics in a self-consistent manner, preserving the hybrid system’s stability.

A key aspect of the interaction is the proper coupling of classical and quantum components. The classical variables influence the quantum system “parametrically”, appearing as externally controlled parameters in $H_{q}+H_{\mathrm{int}}$, while the quantum system influences the classical dynamics through expectation values in the classical equations of motion. This avoids mathematical inconsistencies, yielding a physically valid quantum–classical hybrid system.

Furthermore, $H_{\mathrm{int}}$ introduces an intrinsic feedback mechanism. For example, the term $\lambda q{\hat{\sigma }}_{x}$ leads to continuous bidirectional coupling: The classical displacement *q* modifies the quantum Hamiltonian, altering quantum evolution, while the quantum expectation $\langle {\hat{\sigma }}_{x}\rangle$ contributes to the classical force, modifying the oscillator’s motion. In principle, delayed feedback could be introduced by incorporating memory-dependent terms such as $H_{\mathrm{int}}\left(q\left(t-\tau \right),p\left(t-\tau \right),{\hat{\sigma }}_{x}\right)$, leading to non-Markovian corrections.

### 4.3. Classical Interfaces and Security

In the classical domain, such as computing and communication systems, security at the classical–classical interface, where two distinct classical systems interact, has been extensively studied and developed in both the physical [18] and computational domains [19,20].

Understood as a physical exchange, a classical–classical interface is an exchange of signals or energy between two systems that can be modeled using the tools and intuitions of classical physics. Explicitly, we would say that these signals are reproducible to an arbitrary degree of precision, that the measurement of these signals is *non-destructive*, and that any observed non-deterministic dynamics are the result of incomplete knowledge about the system. The attribute of security, then, pertains to the measurable, deterministic properties of the signal exchange and efforts by attackers or defenders to disrupt or maintain these properties. This covers a wide field of study, from traditional jamming to sensor spoofing and side-channel techniques.

While the term “classical computation” is still somewhat informal, we define a “classical computer” as one that is implementable through a combination of classical states exhibiting deterministic behavior. A classical computer reflects classical physics in its operational logic and behavior. Ideally, it is deterministic, fully traceable, and arbitrarily precise. In a digital system, *arbitrary precision* is mediated by the allocation of discrete resources, i.e., bits, processor cycles, etc. Any ideal interface between two such systems will exhibit similar features, as will the semantics of any symbols exchanged via such an interface.

Because these interfaces convey signals that encode semantically meaningful statements, classical–classical interface security emphasizes techniques that prevent the malicious reading, destruction, or editing of these statements or their constituent symbols. To carry this out, security teams rely on well-established cryptographic methods to encrypt messages, detect tampering, and verify message properties and authorship [19]. Even with crytographic protocols, classical–classical interfaces are vulnerable to certain attacks that exploit the boundaries between systems. For instance, man-in-the-middle attacks, command injection, and protocol and serialization exploitation can all occur during data exchanges between two classical systems, emphasizing the rather plain fact that any shared boundary introduces potential security weaknesses [21]. This understanding of classical–classical interface security offers a valuable perspective when addressing the complexities of the QCI, where traditional methods are insufficient and new security paradigms such as entropy-based measures are required.

### 4.4. Quantum–Classical Interface (QCI)

To formally define the QCI, we wish to consider a general and abstract way to mathematically formulate the QCI. We introduce entropic measures as core mathematical tools to capture information dynamics across QCI, essential in characterizing how information transforms and potentially degrades when transitioning between quantum and classical systems. Beginning with the systems and states of QCI, let Q denote the set of quantum states, i.e., a quantum system, with respect to its underlying Hilbert space H:

(9) $$Q=\{|\psi \rangle \in H:\left[|\psi \rangle \right]=\left\{e^{i\theta }|\psi \rangle \right\}\;\mathrm{and}\;∥|\psi \rangle ∥\}=1\},$$

where [|ψ〉] defines an equivalence class of vectors differing only by a global phase factor $e^{i\theta }$, which ensures phase invariance. Let C denote the set of classical states, i.e., the set of all probability distributions, with respect to its underlying phase space P, which represents all possible configurations of the system:

(10) $$C=\{x:P\to \left[0,1\right]:\int_{P}xd\mu =1\},$$

where dμ is a measure on P; *x* typically represents a probability density over P; we assume the condition that x≥0, and $\int_{P}xd\mu =1$ ensures that *x* is indeed a valid probability distribution. Transformations between the systems are defined via operators, i.e., $T_{QC}:H\to P$, corresponding to the quantum to classical transformation such that $x=T_{QC}(|x\rangle )$. Similarly, $T_{CQ}:P\to H$ corresponds to the classical to quantum transformation such that $|x\rangle =T_{CQ}\left(x\right)$. In practice, when we identify quantum states with density matrices, the $T_{CQ}$ operator takes a vector *x* and maps it to the matrix with diagonal entries that are precisely the vector x. Conversely, the $T_{QC}$ operator takes the density operator and throws away all the non-diagonal entries, leaving just a classical vector. To describe the dynamics of the QCI, we require an operator $L_{QCI}$ that acts on the combined state of the quantum and classical system. While $L_{QCI}$ takes a specific form for a given physical scenario, here, it suffices that it captures a measurement interaction (including the subsequent classical outcome).

As a conceptual framework to capture how classical systems (and their evolution) can influence and be influenced by quantum systems, at the interface where the two meet, i.e., the QCI, we introduce the unitary evolution operator U(t) for the quantum system, and let F(t) denote the time evolution of the classical system. At the QCI, the dynamics is given by the following:

(11) $$U\left(t\right)|\psi \rangle =F\left(t\right)\circ L_{QCI}\circ T_{CQ}\left(x\right),$$

where $T_{CQ}\left(x\right)$ describes the transformation of some classical state *x* into a quantum state: that is, the process of embedding classical information into the quantum domain. Subsequently, the QCI can further influence or transform this quantum state (via the linear operator $L_{QCI}$). Thereafter, the quantum state undergoes its intrinsic time evolution influenced by its interaction with the classical system via F(t) so that the final evolved quantum state can be produced by the left-hand side.

A key aspect of the QCI is the process of measurement [3], where a quantum state collapses to a particular outcome, which is modeled as follows:

(12) $$P_{m}|\psi \rangle =\delta_{m}\left(x\right)T_{QC}(|\psi \rangle ),$$

where $P_{m}$ is the projection operator for a measurement outcome *m* and a corresponding classical indicator function $\delta_{m}\left(x\right)$. Thus, when a quantum state |ψ〉 undergoes a measurement resulting in outcome *m*, it can be represented or interpreted classically as *x* through the transformation $T_{QC}.$

In practice, projective measurements are difficult to implement on hardware due to noise. To address this challenge, we adopt quantum instruments—a general framework that extends the concept of projective measurements to account for realistic, potentially noisy measurement processes, as well as the system’s evolution after measurement. A quantum instrument is mathematically a collection of completely positive trace-non-increasing maps $\left\{E_{k}\right\}$ on the set of density operators D(H) of a Hilbert space H satisfying

(13) $$\sum_{k}E_{k}\left(\rho \right)=E\left(\rho \right),$$

where E is a completely positive trace-preserving map. Each $E_{k}$ represents a possible outcome indexed by *k*, and it determines the post-measurement state conditioned on the outcome *k*. Intuitively, quantum instruments proved a mathematically rigorous way to describe realistic measurements incorporating noise and decoherence; post-measurement dynamics, i.e., how the quantum state evolves after an outcome is observed; and open quantum systems, which model interactions between quantum systems coupled to their environments.

The intrinsic complexities arising from the coexistence of quantum and classical domains in QCI includes decoherence [22], which we have not discussed. However, interactions with a classical environment are a pivotal aspect at this interface. These interactions with the environment can often challenge the preservation of quantum properties. An example might be a qubit interfacing with a classical reading device, highlighting the bidirectional influence of both systems. The task of reliably bridging quantum and classical systems is challenging, with the added difficulty of carrying this out within a set of security measures and constraints, which underscores the significance of our discussions. Furthermore, we not only consider the measurement interactions but also other interactions at the QCI, such as quantum–classical correlations or the challenges arising from the measurement process and classical readout. This also extends to coupling with external forces. Having defined the basic dynamics of the QCI, we now proceed with examining the QCI entropy.

### 4.5. A QCI Example: Classical and Quantum Langevin Equations

To better guide our QCI discussions, we use both a classical and a quantum system, each governed by their respective Langevin equations [23,24,25,26,27]. A QCI is formed through coupling conditions that link these systems and facilitate interaction (see Equation (5)). We first consider a classical system characterized by the position x(t) and velocity v(t) of a particle immersed in a thermal bath. The classical Langevin equation is as follows:

(14) $$m\frac{dv(t)}{dt}=-\gamma v\left(t\right)+F_{e}\left(t\right)+\eta \left(t\right),$$

where *m* is the particle’s mass; γ represents dissipation due to the environment; $F_{e}\left(t\right)$ is an external deterministic force applied to the system; η(t) is a random force modeling thermal noise, with properties 〈η(t)〉=0 and $\langle \eta \left(t\right)\eta \left(t^{\prime }\right)\rangle =2D\delta \left(t-t^{\prime }\right)$, for a noise strength *D*. The state of the system is represented by $u_{1}\left(p_{1}\right)=\left(x\left(t\right),v\left(t\right)\right)$, with $p_{1}$ encompassing parameters such as *m*, γ.

Next we consider a quantum system described by the operator $\hat{a}\left(t\right)$ (e.g., the annihilation operator for a quantum harmonic oscillator) interacting with a quantum environment. The quantum Langevin equation [25] is as follows:

(15) $$\frac{d\hat{a}\left(t\right)}{dt}=-i\omega \hat{a}\left(t\right)-\Gamma \hat{a}\left(t\right)+\hat{\xi }\left(t\right),$$

where ω is the natural frequency of the quantum oscillator, Γ is the damping rate characterizing energy dissipation, and $\hat{\xi }\left(t\right)$ is a noise operator from the environment modeled as a quantum noise source with $\langle \hat{\xi }\left(t\right)\rangle =0$ and commutation relations $\left[\hat{\xi }\left(t\right),{\hat{\xi }}^{†}\left(t^{\prime }\right)\right]=\delta \left(t-t^{\prime }\right)$. The quantum state of the system is represented by $u_{2}\left(p_{2}\right)=\hat{a}\left(t\right)$, with $p_{2}$ containing parameters such as ω, Γ, and properties of the quantum noise $\hat{\xi }\left(t\right)$.

The QCI is established by coupling conditions that link the states of the classical and quantum systems, for example, as follows. The external force $F_{e}\left(t\right)$ in the classical Langevin equation is assumed to be influenced by the quantum system’s observable $\langle \hat{a}\left(t\right)+{\hat{a}}^{†}\left(t\right)\rangle$ (e.g., describing the oscillation amplitude of the quantum state). This creates the following coupling condition:

(16) $$F_{e}\left(t\right)∼g\langle \hat{a}\left(t\right)+{\hat{a}}^{†}\left(t\right)\rangle ,$$

where *g* is a coupling constant. Similarly, the quantum–classical noise operator $\hat{\xi }\left(t\right)$ can be assumed to be influenced by the classical term v(t):

(17) $$\hat{\xi }\left(t\right)={\hat{\xi }}_{0}\left(t\right)+hv\left(t\right),$$

where ${\hat{\xi }}_{0}\left(t\right)$ is the intrinsic quantum noise operator, and *h* is a coupling constant. Such a coupling could be due to back-action, where the classical system affects quantum noise. Combining these interactions, we define the following interface condition:

(18) $$K\left(u_{1},u_{2};p_{1},p_{2}\right)=\left(F_{e}\left(t\right)-g\langle \hat{a}\left(t\right)+{\hat{a}}^{†}\left(t\right)\rangle ,\;\hat{\xi }\left(t\right)-\left({\hat{\xi }}_{0}\left(t\right)+hv\left(t\right)\right)\right)=0,$$

which describes the coupling between the classical and quantum systems, establishing a QCI. Note that our formal interface defined via K is to capture the presence or absence of interactions at the QCI. When K=0 includes non-zero coupling terms, it indicates active interaction and potential for information exchange. When the coupling terms are zero, the systems are independent, and no interaction occurs, and K=0 leads to separate, uncoupled dynamics.

## 5. Entropic Approach to QCI Security

Quantum systems can be susceptible to quantum attacks (e.g., quantum eavesdropping in QKD [28]) or classical attacks on their classical components. The QCI serves as the junction where quantum and classical signals are transduced, and thus, information representation exchanges occur, posing security challenges that are countered by monitoring the entropy at the QCI, offering a way to characterize potential information leaks.

### 5.1. Classical Entropy

Classical information theory invokes entropy to quantify and analyze uncertainty and information in various communication systems. For system C, the measure of uncertainty for a discrete random variable *X* with possible outcomes ${\left\{x_{i}\right\}}_{i}$ from a finite alphabet X and corresponding probability distribution ${\left\{p_{i}\right\}}_{i}={\left\{p\left(x_{i}\right)\right\}}_{i}$ is the realization that $x_{i}$ occurs, and this is given by the Shannon entropy defined as follows:

(19) $$H\left(X\right):=-\sum_{i=1}^{n}p\left(x_{i}\right)\log p\left(x_{i}\right),$$

with the base of the logarithm taken to be 2 to measure H(X) in bits. For more information about Shannon entropy, its applications, and its mathematical characteristics, see Chapter 10 in [29].

**Example 2.**  *From the example in Section 4.5, recall that the classical system is a particle in a thermal bath described by the Langevin equation. If the system reaches thermal equilibrium, the probability distribution $P(u_{1})$ follows a Boltzmann–Gibbs distribution:*

(20) $$P\left(u_{1}\right)=\frac{1}{Z}\exp\left(-\frac{E(x,v)}{k_{B}T}\right),$$

*where T is the temperature, $k_{B}$ is Boltzmann’s constant, $E\left(x,v\right)=\frac{1}{2}\left(mv^{2}+kx^{2}\right)$ is the total energy of the system, and $Z=\frac{2\pi k_{B}T}{\sqrt{km}}$ is the normalization factor. To simplify the calculations, we divide the phase space into small bins so that the classical state is $u_{1}=\left(x,v\right)$. Thus, each probability is $p\left(x_{i}\right)\propto \exp\left(-\frac{E(x,v)}{k_{B}T}\right)$. With this, one could calculate the Shannon entropy as in Equation (19).*

### 5.2. Quantum Entropy

In the case of Q, the von Neumann entropy *S* similarly describes the concept and quantification of uncertainty or the “entropy” associated with the quantum state or its density representation, ρ, of the quantum system. Its definition is analogous to the Shannon entropy for C:

(21) S(ρ)=−Tr(ρlogρ),

with the logarithm in base 2 to express S(ρ) in bits. For more information about von Neumann entropy, its applications, and its mathematical characteristics, see Chapter 11 in [29].

**Example 3.**  *The quantum system above is also a harmonic oscillator coupled to a thermal bath at temperature T. The density operator in thermal equilibrium is*

(22) $$\rho =\frac{1}{Z}\exp\left(-\frac{\hat{H}}{k_{B}T}\right),$$

*where $\hat{H}=\hbar \omega \left({\hat{a}}^{†}\hat{a}+\frac{1}{2}\right)$ is the Hamiltonian.*

### 5.3. QCI Entropy

At the QCI, entropy features both quantum and classical elements, providing an “interfacial” measure of uncertainty. The QCI system, denoted as $\rho_{QC}$ (a tensor product of quantum and classical states), and its entropy $S_{QC}$ capture the collective uncertainty of both domains. This encompasses not only the individual uncertainties of Q and C but also the correlations and coherence established at the interface. Consequently, QCI entropy can be interpreted in two complementary ways: (1) as the total entropy of the joint system and (2) as a composite entropy that dissects individual contributions and interactions between quantum and classical subsystems. More formally, consider the following quantum–classical ensemble:

$${\left\{p_{C}\left(x\right),\rho_{Q}^{x}⊗|x\rangle {\langle x|}_{C}\right\}}_{x\in C}\;,$$

where the first system (*Q*) is a quantum system, and the second system (*C*) is classical system. The correlation between the quantum and classical state is represented by associating the classical state |x〉 with each quantum density operator $\rho_{Q}^{x}$. Here, ${\left\{|x\rangle \right\}}_{x\in C}$ forms an orthonormal basis for the classical subsystem. The bipartite state of the quantum–classical system is expressed as follows:

$$\rho_{QC}=\sum_{x\in C}\rho_{Q}^{x}⊗p_{C}\left(x\right)|x\rangle {\langle x|}_{C}.$$

In this setup, the classical state *C* is perfectly distinguishable. In this case, the joint entropy is given by

(23) $$S\left(\rho_{QC}\right)=H\left(C\right)+\sum_{x\in C}p_{C}\left(x\right)S\left(\rho_{Q}^{x}\right),$$

where H(C) is the classical entropy of a random variable C; $p_{C}\left(x\right)$ is in terms of the measurement outcomes, namely, $p_{C}\left(x\right)=\mathrm{Tr}\left(\rho_{Q}M_{x}\right)$, for a given positive-operator valued measure associated with the classical variable *x*. eq:qc entropy quantifies the total uncertainty or lack of information about the combined quantum–classical system; however, it does not indicate which part of the system the uncertainty is coming from. For this, we use a mutual information measure:

(24) $$I\left(Q:C\right):=S\left(\rho_{Q}\right)+H\left(C\right)-S\left(\rho_{QC}\right).$$

Mutual information quantifies the dependency or correlation between *Q* and *C*. Unlike joint entropy, which globally measures uncertainty, mutual information focuses on the shared content between subsystems and thus highlights how the classical subsystem “leaks” information into or about the quantum system and vice versa.

### 5.4. Information Flow at QCI

Information flow refers to how data moves within a system, between systems, or across different security domains. When a quantum system is measured, as described by a set of positive-operator valued measures $\left\{M_{i}\right\}$, the probability of outcome *i* is as follows:

(25) $$p\left(i\right)=\mathrm{Tr}\left(M_{i}^{†}M_{i}\rho \right).$$

This transformation from ρ to p(i) represents the QCI. The entropy across this boundary, i.e., transformation between classical and quantum systems or vice versa, can be viewed as a change in information when transitioning from a quantum to a classical system. This change is typically represented by a change in entropy. The choice of entropic function here depends on the domain interest and is chosen from the mentioned entropic functions in this section. Furthermore, the QCI acts as a bridge for information exchange between Q and C.

#### 5.4.1. Quantum-to-Classical (H→P)

In the quantum context, information flow primarily pertains to the measurement process: a measured quantum state yields a specific outcome that can be recorded classically. Through this flow, the transition is facilitated by $T_{QC}$, which deciphers the result of quantum computation or quantum systems’ evolution.

#### 5.4.2. Classical-to-Quantum (P→H)

Conversely, this flow direction involves quantum-state prep based on classical data or instructions, e.g., during the initialization of quantum systems, where classical data might be encoded into a quantum form, represented by the transformation $T_{CQ}$.

#### 5.4.3. Interplay at QCI

At the QCI, quantum and classical states coexist and interact simultaneously. For instance, while a quantum algorithm runs, classical controls might adjust parameters based on intermediate quantum states. In this discussion, such intertwined dynamics necessitates a comprehensive understanding of information management and exchange at the QCI, ensuring reliable operation.

### 5.5. Inferring Information Flow Using Entropy at the QCI

One primary concern at the QCI is decoherence [22], where quantum information is lost to its environment, which is typically classical in nature. This loss is not just a transfer of information but an unwanted leakage that may change entropy $S_{QC}$. When the quantum system interacts with an external environment (i.e., an open quantum system) or when noise is present, the system undergoes decoherence. This leads to an evolution in its state, resulting in a new density matrix, $\rho^{\prime }$, often determined using quantum master equations. The change in entropy due to such interactions can be quantified by the following relative entropy:

(26) $$S(\rho ||\rho^{\prime }):=\mathrm{Tr}\left(\rho \log\rho -\rho \log\rho^{\prime }\right).$$

Although $S(\rho ||\rho^{\prime })$ itself does not reveal the direction of information flow, the change in entropy can provide useful metrics. In particular, relative entropy is useful for quantifying how much the system has deviated from its original state ρ to its decohered state $\rho^{\prime }$. When $S(\rho ||\rho^{\prime })$ is closer to 0, this signifies very little change from ρ to $\rho^{\prime }$. Conversely, if $S(\rho ||\rho^{\prime })$ is large, this indicates that a major change in the system has occurred. A decline in $S(\rho ||\rho^{\prime })$ post-measurement points toward a quantum-to-classical information flow. Such measurements involve classical control signals, used to either manipulate or initialize quantum states. If a classical control steers the quantum system into a more certain (less mixed) state, this transition might represent information flow from the classical domain to the quantum one. A scenario closely related to this is quantum feedback [4,30]: A quantum system is measured, the outcome (now classical) is processed, and then a classical control signal is relayed back to influence the quantum system, suggesting a cyclical information flow: first from the quantum to classical system (through measurement) and then from the classical system back to the quantum system (via control).

### 5.6. Quantum Phenomena and Their Influence on QCI Entropy

Consider a quantum state |ψ〉 of Q in a *d*-dimensional Hilbert space:

(27) $$|\psi \rangle =\sum_{i=1}^{d}c_{i}|i\rangle ,$$

where $|c_{i}{|}^{2}$ represents the probability of finding the system in state |i〉, with each being orthogonal to one another. In general, the entropy *S* of a state is given by

(28) $$S=-\sum_{i=1}^{d}|c_{i}{|}^{2}\log_{2}{\left|c_{i}\right|}^{2},$$

That is, the superposition’s inherent uncertainty contributes to the entropy of quantum states.

For example, consider the Bell state (a canonical example of an entangled state for two qubits):

(29) $$|\Phi^{+}\rangle =\frac{1}{\sqrt{2}}(|00\rangle +|11\rangle ),$$

for which entropy takes on a particularly intriguing role. The joint system, described by the Bell state for instance, exhibits zero entropy when considered as a whole. However, when considering each qubit (or subsystem) separately, entropy is maximized. As quantum states decohere, they tend to evolve into mixed states. A pure quantum state has well-defined entropy based on its superposition coefficients. As decoherence progresses, the off-diagonal elements of the system’s density matrix decrease, leading to an increase in entropy until the system is fully decohered.

An entropy-based security framework for the QCI should establish conditions where entropy fluctuations indicate vulnerabilities. A formal theorem or statistical threshold can enable anomaly detection by flagging significant deviations from a baseline. Such thresholds would depend on the applications and materials used. Practical hybrid quantum–classical systems, including quantum computing, QKD, and quantum sensors, provide testbeds for studying entropy dynamics [31,32]. Moreover, security assessments must account for adversarial strategies like side-channel exploitation, covert entropy leaks, and decoherence injection to distinguish genuine threats from environmental noise [33,34]. Such concepts would require additional investigations for assessments and standardization.

To highlight the implications of entropic measures, we return to QKD. Monitoring fluctuations in entropy can detect unexpected changes in information leakage patterns, potentially revealing side attacks or malicious interferences. Similarly, in hybrid control systems that use classical controllers to adjust quantum parameters, entropy anomalies can reveal subtle injections of spurious classical commands. Additionally, entropy can play a role in embedded quantum security modules within classical networks. For example, by continuously monitoring the entropy outputs of hardware-based encryption keys, one can detect manipulation. While these examples illustrate the promising applications of entropy-based metrics at the quantum–classical interface, real-world deployment remains limited due to the technical and infrastructural challenges of constructing scalable, noise-resilient quantum hardware.

## 6. Other Metrics

In our modular approach, the presented entropy-based metric could be broadened by aligning it with elements from the statistical estimation theory, such as Fisher information and the Cramér–Rao bound [35,36,37], leading to a higher degree of the conceptual and quantitative assessment of the QCI’s sensitivity to attacks or anomalies. Adding a statistical layer helps define the boundaries within which entropy or mutual information can be meaningfully detected at the QCI. By establishing thresholds for detectable entropy or mutual information, the statistical layer defines where quantum effects are distinguishable from classical contributions, clarifying the operational boundaries of the QCI.

To formally discuss parameter estimation in both classical and quantum settings, we define both the classical and quantum Fisher Information and the corresponding Cramér–Rao Bounds [37,38]. For a random variable *X* with probability density function p(X|θ) that depends on an unknown parameter θ, *classical Fisher information*I(θ) is defined as follows:

$$I\left(\theta \right):=E\left[{\left(\frac{\partial }{\partial \theta }\ln p\left(X|\theta \right)\right)}^{2}|\theta \right],$$

where lnp(X|θ) is the logarithm of the likelihood function; $\frac{\partial }{\partial \theta }\ln p\left(X|\theta \right)$ is the score function, which measures the sensitivity of the likelihood to changes in θ; and E[·|θ] denotes the expectation taken with respect to the distribution p(X|θ). Note that I(θ)≥0 because the term is squared. Moreover, the dependency of θ ensures that $\frac{\partial }{\partial \theta }\ln p\left(X|\theta \right)$ is generally non-zero for some values of *X*. This ensures that I(θ) is non-zero and hence well defined.

Classically, the Cramér–Rao bound states that for any unbiased estimator $\hat{\theta }$ of θ, the variance of $\hat{\theta }$ is bounded by the reciprocal of the Fisher information:

$$\mathrm{Var}\left(\hat{\theta }\right)\geq \frac{1}{I(\theta )},$$

which indicates that the variance of any unbiased estimator cannot be lower than $\frac{1}{I(\theta )}$, setting a fundamental limit on estimation precision.

The *quantum Fisher information* $I_{Q}\left(\theta \right)$ is defined as follows:

$$I_{Q}\left(\theta \right)=\mathrm{Tr}\left(\rho_{\theta }L_{\theta }^{2}\right),$$

where $L_{\theta }$ is the symmetric logarithmic derivative (SLD) with respect to θ. The SLD is defined implicitly by the following:

$$\frac{\partial \rho_{\theta }}{\partial \theta }=\frac{1}{2}\left(\rho_{\theta }L_{\theta }+L_{\theta }\rho_{\theta }\right),$$

where $L_{\theta }$ depends on the state $\rho_{\theta }$ and encodes the sensitivity of the quantum state to changes in θ.

Similarly, the quantum Cramér–Rao bound states that for any unbiased estimator $\hat{\theta }$ of the parameter θ based on measurements on the quantum state $\rho_{\theta }$, the variance is bounded by the reciprocal of the quantum Fisher information:

$$\mathrm{Var}\left(\hat{\theta }\right)\geq \frac{1}{I_{Q}\left(\theta \right)},$$

which represents the ultimate limit on estimation precision imposed by the quantum nature of the system, and it is generally tighter (i.e., more precise) than the classical Cramér–Rao bound for measurements on the quantum state [39].

## 7. Criteria for QCI Security

Our objective here is to provide a perspective on using entropy changes at the QCI as a potential metric for evaluating security concerns. We use a simple definition of security, namely, that the system only does what it is designed to do and nothing more. The idea of using entropic functions to quantify uncertainty allows us to more formally quantify instances in security where "more occurs than a given system is expected to do". Given a quantum system Q and a classical system C, interfacing at the QCI, the cumulative entropy across the QCI is given by eq:qc entropy.

The concept that any significant, unexpected change in $S\left(\rho_{QC}\right)$ could potentially be an initial indication of a security breach or an anomaly is what we aim to elaborate. This concept posits that while individual changes in the entropies of Q or C might be expected due to the normal dynamics of each system, it is the combined entropy across the interface that serves as a reliable measure of system integrity. If a theorem can be devised as a theoretical foundation, its practical implications could prove useful.

Entropy measurements of this kind can form the basis of (or at least inform) a framework for identifying QCI operational security criteria, characterizing nominal dynamics, locating vulnerabilities, and flagging suspicious QCI behavior. Ideally, our proposed entropy measurements would be sensitive to a wide range of cyber incident or system failure types, and they may be useful for detecting the following:

1. Information leakage;
2. Anomalies in mutual information at the QCI, indicating unintended data transfer;
3. Consistency in entropic dynamics;
4. Assessments of relative entropy between expected and observed values (states) reveal unexpected perturbations, suggesting possible data injection or tampering;
5. Control entropy decreases via data processing.

### Classical Security Approaches

This approach, while promising, faces significant practical and conceptual challenges. Real-world quantum systems, when interfacing with classical components, often operate under noisy conditions. In such scenarios, distinguishing between routine entropy fluctuations and potential threats becomes crucial.

Furthermore, all but the simplest information systems, be they quantum or classical, are fundamentally discontinuous. Consider a simple finite-state machine defined by the following tuple:

(30) θ=(Q,i,F,Σ,δ),

where *Q* is the set of system states, *i* the initial state, *F* the terminating state, Σ the input alphabet, and δ the transition function such that Q×Σ→Q. The input alphabet consists of all possible three tuples over the symbol set {A,B,C}; however, not all alphabet tuples will result in a state transition given the machine’s current state $q_{n}\in Q$ and the components of δ.

Assume that the following alphabet symbol μ=CAB initiates the following state transition:

(31) $$\hat{B}\left(\mu ,q_{1}\right)=\left(q_{1}\to q_{2}|q_{1},q_{2}\in Q\right),$$

which is permitted under *Q*, Σ, and δ, but this violates a specified security policy. In this scenario, a state-agnostic, entropy-based anomaly detector is likely to fail to flag this illegal transition, as it cannot differentiate it from benign inputs that yield similar entropy. Specifically,

(32) $$H(\hat{B}\left(\mu ,q_{1}\right)=H\left(B_{legal}\left(\sigma \left(\mu \right),q_{i}\right)\right),$$

where σ(μ) is any permutation of the input symbol μ. In other words, even in completely noiseless systems, arbitrarily similar input strings can produce qualitatively different behaviors in a given information system, but they yield identical Shannon entropy calculations.

Any operation across a QIS QCI interface consists of the transmission or transduction of information symbols. As we have shown, insofar as our proposed use of entropy is used *solely* to naively characterize symbols or simple operations, its utility will probably be limited. To make entropy useful, it must be correlated with the system-level analyses and real-time measurements of the larger QIS.

Classical cybersecurity suggests several methods for carrying this out. The **system analysis** approaches include the following:

1. **Threat modeling**: Identify, rank, and describe cyber threats by analyzing relationships between business or organizational risk, threat actor models, and system weaknesses [40]. This process provides a high-level context for the security needs, location, and disposition of QCIs.
2. **Static/formal analysis**: Locates potential vulnerabilities by programmatically analyzing system specifications or the source code [41]. This general technique could be modified to identify high-sensitivity QCIs and describe their expected entropy dynamics.

Other approaches more directly modify or monitor the QIS. These **system monitoring** approaches include the following:

1. **Digital twin**: Compare measurements of the QIS with a system-state-aware computational model. A divergence in QCI entropy can be compared in real-time to other system variables, allowing for rapid fault diagnosis.
2. **Integrated countermeasure**: Analyze and rewrite the quantum program to create QCIs where entropy dynamics are easier to profile or measure.

To the best of our knowledge, these mitigation strategies remain largely unexplored in practical quantum systems.

## 8. Discussions

The quantum–classical Interface (QCI) theory benefits from a multi-layered approach, balancing granular and comprehensive perspectives to effectively analyze system security.

A granular perspective focuses on the expectation values of individual observables, enabling the identification of specific vulnerabilities. By isolating the impact on particular observables, this approach allows for precise troubleshooting, making it ideal for detecting localized disturbances and pinpointing minute vulnerabilities in the system. This approach, however, can sometimes overlook broader, holistic changes in the system that emerge from interactions across multiple observables or collective behavior. Additional complexities arise due to repeated tampering, which, if occurring at certain intervals, might resonate with quantum dynamics, amplifying the effect on potential thresholds.

Conversely, a comprehensive perspective aims to capture the overall state integrity by examining global indicators like entropy changes. By monitoring several entropic functions, the system can encapsulate global disturbances, shifts in information flow, and the influence of substantial perturbations on the system as a whole. A large perturbation, in this context, typically implies a noticeable difference between the tampered and untampered states, informing about potential security breaches and information leakage on a macroscale. However, this broader scope may miss subtle disturbances, especially those confined to specific parts of the system.

In practical applications, a combined use of these perspectives could offer both a global overview and detailed diagnostics. By adapting the approach to fit the system’s unique features and security needs, one could maintain a comprehensive understanding of overall system health through entropy measurements while also addressing specific vulnerabilities at the observable level. This combined strategy could provide a versatile and effective framework for managing the diverse security challenges within quantum–classical systems.

It is important to acknowledge limitations in entropy-based methods. In high-noise environments or thermally fluctuating systems, entropy changes may be dominated by benign physical dynamics, making it difficult to distinguish attack signatures. Moreover, entropy alone does not indicate causality or intent. For these reasons, entropy metrics should be viewed as complementary to structural or rule-based security tools. Integrating entropic diagnostics with baselining models and contextual monitoring would yield a more robust hybrid security framework.

## 9. Conclusions

This position paper introduces an entropy-based framework for understanding and securing the QCI. By outlining essential security criteria and potential vulnerabilities, we aim to catalyze ongoing discussions and encourage empirical studies that will further solidify and expand QCI security strategies in emerging quantum technologies. Our work emphasizes the significant need of QCI entropy in the broader context of quantum technology development.

## Data Availability

The original contributions presented in this study are included in the article. Further inquiries can be directed to the corresponding author.

## Abbreviations

The following abbreviations are used in this manuscript:

ORNL	Oak Ridge National Laboratory
QCI	Quantum–classical interface
